# Neonatal Hyperbilirubinemia in a Marginalized Population on the Thai-Myanmar Border: a study protocol

**DOI:** 10.1186/s12887-017-0798-8

**Published:** 2017-01-21

**Authors:** Laurence Thielemans, Margreet Trip-Hoving, Germana Bancone, Claudia Turner, Julie A. Simpson, Borimas Hanboonkunupakarn, Michaël Boele van Hensbroek, Patrick van Rheenen, Moo Kho Paw, François Nosten, Rose McGready, Verena I. Carrara

**Affiliations:** 1 Shoklo Malaria Research Unit, Mahidol-Oxford Tropical Medicine Research Unit, Faculty of Tropical Medicine, Mahidol University, Mae Sot, Thailand; 2 Department of Paediatrics, Université Libre de Bruxelles, Faculty of Medicine, Brussels, Belgium; 3 Centre for Tropical Medicine and Global Health, Nuffield Department of Medicine, University of Oxford, Old Road Campus, Oxford, United Kingdom; 4 Cambodia-Oxford Medical Research Unit, Angkor Hospital for Child, Siem Reap, Cambodia; 5 Angkor Hospital for Children, Siem Reap, Cambodia; 6 Centre for Epidemiology and Biostatistics, Melbourne School of Population and Global Health, The University of Melbourne, Melbourne, Australia; 7 Mahidol-Oxford Tropical Medicine Research Unit (MORU), Faculty of Tropical Medicine, Mahidol University, 420/6 Ratchawithi Rd, Bangkok, Thailand; 8 Global Child Health Group, Emma Children’s Hospital/Academic Medical Center of the University of Amsterdam, Amsterdam, The Netherlands; 9 Department of Pediatric Gastroenterology, University of Groningen, University Medical Center Groningen, Groningen, The Netherlands

**Keywords:** Neonatal hyperbilirubinemia, Jaundice, Phototherapy, Infant, Low-resource, Refugee, Migrant, Resource-limited setting, G6PD deficiency, Neurodevelopment

## Abstract

**Background:**

This study aims to identify risk factors and the neurodevelopmental impact of neonatal hyperbilirubinemia in a limited-resource setting among a refugee and migrant population residing along the Thai-Myanmar border, an area with a high prevalence of glucose-6-phosphate dehydrogenase-deficiency.

**Methods:**

This is an analytic, observational, prospective birth cohort study including all infants of estimated gestational age equal to or greater than 28 weeks from mothers who followed antenatal care in the Shoklo Malaria Research Unit clinics. At birth, a series of clinical exams and laboratory investigations on cord blood will be carried out. Serum bilirubin will be measured in all infants during their first week of life. All the infants of the cohort will be clinically followed until the age of one year, including monitoring of their neurodevelopment.

**Discussion:**

The strength of this study is the prospective cohort design. It will allow us to collect information about the pregnancy and detect all infants with neonatal hyperbilirubinemia, to observe their clinical response under treatment and to compare their neurodevelopment with infants who did not develop neonatal hyperbilirubinemia. Our study design has some limitations in particular the generalizability of our findings will be limited to infants born after the gestational age of 28 weeks onwards and neurodevelopment to the end of the first year of life.

**Trial registration:**

ClinicalTrials.gov ID NCT02361788, registration date September 1st, 2014.

## Background

Clinically significant neonatal hyperbilirubinemia (NH) is an important cause of neonatal morbidity and mortality and the most frequent reason for delayed hospital discharge or readmission in the first week of life worldwide [1, 2]. Low- and middle-income countries, however, disproportionately bear the burden of severe NH [1, 3] especially countries with a high prevalence of glucose-6-phosphate dehydrogenase (G6PD)-deficiency [4]. In addition to the difficulty of establishing routine G6PD-deficiency screening in newborns as recommended by the World Health Organisation [5] other challenges which impact on NH prevail, including: rhesus sensitization, maternal health-seeking behaviour, availability of neonatal units, and use of guidelines for early recognition and treatment of NH. In developing countries, NH is also one of the main causes of neurodevelopmental impairment in infants, especially in premature infants [1, 3], even though the harmful consequences of NH could be easily prevented by early detection and phototherapy [6, 7].

A recent meta-analysis of NH in low- and middle-income countries highlighted the need for more robust epidemiological studies to identify additional risk factors that may be particular to these settings [3]. In addition, there are no guidelines to prevent and manage NH in low resources settings. Olusanya, et al. have proposed adapting existing guidelines from high level income countries for NH screening in low resource settings [8]. However, as NH is often the result of complex interactions with multiple causes linked to the population and the environment, the development of context-specific guidelines may be more appropriate for these populations particularly affected by NH.

This manuscript summarizes the protocol of an analytic, observational, prospective birth cohort study which will identify local risk factors associated with NH in a limited-resource setting. As far as we are aware this will be the largest birth cohort study that combines research on the risk factors of NH and follow-up monitoring up to one year of life in South East Asia [9–12]. The information gathered from this study will be used to improve and adapt existing guidelines for resource-constrained environments, help develop locally relevant protocols, and make evidence-based recommendations on where and how limited resources can be best allocated to reduce the risks and the negative outcomes of NH. At the time of writing this protocol seven of the eight refugee camps on the Thai-Myanmar border where the study will take place do not have access to point of care G6PD testing, serum bilirubin (SBR) measurement or phototherapy units.

## Methods

### Objectives

The study primary objective is to determine the risk factors associated with moderate and severe NH [6]. The secondary objectives are to: i) determine the incidence of NH and of prolonged jaundice; ii) compare the SBR trend and response to phototherapy by risk factors associated with moderate and severe NH; iii) determine whether severe NH impairs neurodevelopment.

### Setting

The Shoklo Malaria Research Unit (SMRU) is a recognised field research unit working with the refugee (since 1986) and the migrant populations (since 1998) residing on the Thai-Myanmar border [13]. SMRU provides free medical and obstetric care in three clinical sites along the border. All women are invited to the antenatal care (ANC) clinic as soon as they are aware of their pregnancy. During the first ANC visit a dating ultrasound accurately determines the gestational age of the pregnancy. This is important in the context of this study because prematurity is a major risk factor for NH [6]. All women attending ANC are routinely screened for G6PD (fluorescent spot test) and thalassemia, and their ABO group is determined. They are provided with maternity services and are encouraged to deliver at the SMRU clinic although traditionally women birthed at home. Neonates in need of medical attention are admitted to the special-care baby unit (SCBU) and older infants to the inpatient department.

The study will take place in the three SMRU-SCBUs (established between 2008 and 2009) where local medics and nurses work under supervision and receive training by an expatriate doctor. SBR measurement and blue light phototherapy were introduced in 2009 using the US National Institute for Health and Care Excellence Guidelines (NICE) for clinical care of NH [6].

### Study design and patient recruitment

The study will be an analytic, observational, prospective birth cohort study.

**Fig. 1:**
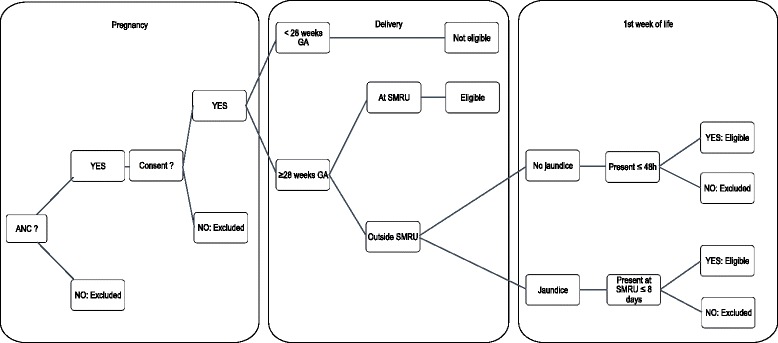
Enrolment and eligibility. The figure presents the enrolment eligibility criteria in pregnancy, at birth (for those born in SMRU settings) and in the first week of life (for those born at home or another hospital)

All women attending ANC will be provided with information about the study during the 3rd trimester of their pregnancy and provide informed consent for the newborn if they are willing to participate. Maternal demographic, medical and obstetric history will be collected at the time of enrolment. All infants born in an SMRU birthing facility will be included unless born extremely premature (defined in this study and other low resource settings as a gestational age of less than 28 weeks). In this cohort, the incidence of NH will be determined (Fig. 1).

Of the women who attended SMRU ANC, approximately 10% give birth at home and 8% in another hospital. Any infant born outside the SMRU clinic will be eligible if they present in the first 48 h of life, or if they present with jaundice from 48 h to 8 days of life.

The study start-up will be staggered to ensure that each of the teams in the three study clinical sites can operate smoothly and consistently. At each site, the period of enrolment will last one calendar year. The infants will be followed up monthly until they reach twelve months of age. The study recruitment is expected to close on May 13, 2017.

### Study procedures

**Fig. 2:**
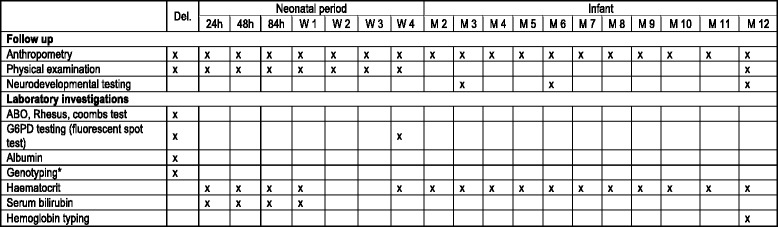
Study procedure timeline. Timeline of the different clinical and laboratory procedures from birth to 1 year of age. * G6PD deficiency, Southeast Asian Ovalocytosis and Gilbert’s syndrome. Del: Delivery, W: week, M: month

A timeline of the study procedures is presented in Fig. 2. At birth, umbilical cord blood will be collected. The use of umbilical cord blood allows enough blood volume for several tests including: blood group and Coomb’s test, G6PD testing, albumin level and genotyping of three different loci (G6PD-deficiency, Southeast Asia Ovalocytosis and Gilbert’s syndrome).

Within the first 24 h a physical examination including anthropometry measurements and clinical jaundice evaluation will be performed as part of routine neonatal care. The mother will be questioned about breastfeeding, use of medicines or preparations for the infant, and the use of naphthalene creams or balls. The NICE Guidelines for clinical care of NH, defining, for each gestational age, SBR measurement and treatment thresholds for moderate and severe NH, will be used in this study to minimize misclassification.

The follow-up schedule will consist of 4 visits in the first week of life, weekly visits until 1 month of age and then monthly visits until 12 months of age (Fig. 2). To ensure adequate attendance to follow-up, each appointment date will be reported in the participant’s medical booklet and parents of the participant who missed an appointment will be called by phone or visited by a health worker. In addition, parents accompanying the child will be compensated for loss of work and travel expenses at an appropriate local rate.

During the visits, routine examination will include anthropometric measurements and physical examination. Laboratory examination will include regular haematocrit (Hct) testing, G6PD testing repeated at 1 month and haemoglobin (Hb) typing at 1 year of age. Quantitative spectrophotometric assay will be carried out in a subsample of females to characterize the enzymatic activity of heterozygous subjects and the associated risk of developing NH.

The infant neurodevelopment will be assessed using the Shoklo Developmental Test at 3, 6, and 12 months [14]. Scoring will be based on a pass/fail system for each item of the test; the global score of the test will be obtained by adding up all the passed scores of the following categories; eye-hand coordination, gross motor, social and speech development. Tone and behaviour during the test will be assessed and scored separately by circling the most appropriate pre-coded diagram (for tone) or description (for behaviour).

### Clinical care

The clinical and research experience of the staff in conjunction with reliable laboratory services and treatment facilities will permit an efficient management of NH. Each SBR measurement will be immediately plotted on a jaundice treatment graph specific for the gestational age (NICE graph) to assess the need for additional follow-up visits, phototherapy, or referral to a tertiary hospital. Infants requiring an exchange transfusion (SBR > 550 μmol/L at any time, with or without clinical signs) will be transferred to the local Thai Hospital in Mae Sot (60 km away). In case of prolonged jaundice (more than 21 days) there will be further investigations [6].

During the neonatal period, weight will be regularly measured and if there is ≥ 10% weight loss compared to the birth-weight the infant will be examined carefully for any underlying causes and necessary care will commence. All medical conditions including anaemia, defined as Hct < 33% will be treated according to standardized guidelines used in this area (Burmese Border Guidelines [15]).

### Outcomes

The primary outcome of this study will be to measure the relative risk of factors associated with moderate and severe NH in infants born at SMRU and to calculate their population-attributable fraction. The secondary outcomes will be to: i) determine the incidence of NH and of prolonged jaundice; ii) measure the changes in the trend of consecutive SBR and the duration of phototherapy according to the risk factors; iiia) compare the clinical condition (anaemia incidence, stunting prevalence) between severe and moderate NH, and normal bilirubinaemia in the first year of life; iiib) compare the neurocognitive developmental, tone and behaviour total scores at one year of life between severe and moderate NH, and normal bilirubinaemia.

### Sample size

A convenience sample including all births within a one year timeframe at each site was chosen. Based on previous SMRU studies, it is assumed that most mothers will agree to participate in this study and that 15% of infants will be lost to follow-up before completion of the study. More than 90% of women in the area attend ANC. In 2014 more than 80% of women who attended ANC care birthed at SMRU clinics (2040 infants born alive of 28 weeks or more gestational age). It is expected that 1734 infants will be included in the study.

With a total sample size of 1734 infants and an approximate prevalence for NH of 15%, the statistical power (two-sided 95% confidence interval) of a relative difference in the risk of NH of 1.5 fold and 2-fold, in association with a binary risk factor with a prevalence of 10, 20 or 50%, is presented (Table 1).

**Table 1:** Statistical power calculations

	Relative risk (RR) for NH associated with binary risk factor	
	RR = 1.5	RR = 2.0
Binary risk factor prevalence	Statistical power	
10%	71%	99%
20%	86%	99%
50%	97%	99%

### Ethical considerations

The population served by the SMRU clinics are predominantly migrant workers and refugees. These individuals have lower levels of income and education, poorer health outcomes, and migratory housing arrangements. SMRU has been serving this population for over 30 years and has experience of working with vulnerable populations. SMRU medics, nurses and health workers are recruited from the same areas as the patient population and are sensitive to their needs. All communication is conducted in the preferred language of the patient. We ensure that participation is voluntary and that it is clearly understood that participation in the study can be ceased at any time without effect to the care provided.

Trained counsellors will seek the mother’s consent after the patient information sheet has been discussed. All queries from the mothers will be answered and those who agree will sign the consent form. Trained personnel carry out all capillary and venous blood taking to reduce infant’s discomfort from these procedures. All data collected will be treated confidentially and stored securely.

### Data collection and analysis

Data will be collected on pre-coded forms and stored in a purpose-designed Microsoft Access database. A hypothesised causal diagram for how factors associated with NH may be connected will be constructed. The incidence of NH and corresponding 95% confidence interval will be calculated within the population of newborns born in the SMRU clinics only; eligible newborns born outside SMRU facilities will be excluded from the incidence analysis as they might falsely increase the incidence of NH in this population. Following this, univariate and multivariate logistic regressions will be performed to identify risk factors for NH. Stepwise logistic regression modelling with backward elimination will be performed on the subset of candidate risk factors selected based on *p*-value < 0.10 in the univariate regression analyses. Key confounders, identified a priori in the causal diagram will be forced into the model. To estimate the association between NH and neurocognitive score, multivariate linear regression will be performed with adjustment for the confounders identified in the causal diagram. Pre-planned subgroup analysis will be conducted in order to identify risk factors associated with moderate and severe NH and estimate the association between NH and neurodevelopment (measured as a continuous neurocognitive score). The description of the clinical course will be supported by SBR measurement tracking recorded on the treatment graph. SBR measurements will be modelled to understand factors impacting on trends [6]. A senior biostatistician will oversee the statistical analyses.

## Discussion

There are several strengths to the proposed protocol. First, it is a prospective birth cohort study. The active screening for NH will help early detection, treatment and severity categorization of the NH. Moreover, it will allow the comparison of neurodevelopmental scores at 1 year of age between infants with and without NH, an important and unique feature of the study.

Secondly, the collection of umbilical cord blood will provide valuable information otherwise rarely available on possible NH predisposing factors, such as the presence of a UGT1A1 polymorphisms (Gly71Arg and TATA promotor) associated with or without Gilbert’s and/or Crigler-Najjar syndrome [16], or Southeast Asian Ovalocytosis [17, 18].

G6PD-testing (fluorescent spot test) performed on the cord blood can be falsely normal because of the high number of reticulocytes and young red blood cells with a higher level of activity or when haemolysis is present [19]. For this reason the fluorescent spot test will be repeated in all infants at the age of one month. This will give valuable information about the reliability of one of the most frequently used screening tests in resource-constrained settings where the test is most often performed only at birth.

G6PD-deficiency is very common among the refugee and migrant populations living on the Thai-Myanmar border [20]. This study will quantify the contribution of G6PD-deficiency to the incidence and severity of NH in these populations. It will confirm clinical experience that a high proportion (25%) of newborns requiring phototherapy are G6PD-deficiency and that these newborns require a longer phototherapy duration [21]. Furthermore it will account for a common practice in these populations [22]: the use of naphthalene creams or balls containing naphthalene stored with infant clothes. There is a risk for the infant of naphthalene skin absorption, inhalation or accidental ingestion, which is a known cause of haemolysis in G6PD deficient patients [23, 24].

As eligible babies are from mothers who followed ANC and the majority of mothers give birth in SMRU facilities, detailed pregnancy information will be available. In Asia, ABO blood group incompatibility and G6PD-deficiency are the two most commonly known causes of NH [1].

On the negative side, women who choose not to attend ANC or are unable to attend will be omitted from this study. These women are known to be at higher risk of complications in labour and their offspring might need more medical attention [25]. Nonetheless there is a possibility for infants born outside the SMRU birth units and presenting within 48 h of life (or up to 8 days of life in infants with NH) to be included in the study and to be followed up until one year of age.

This protocol has some additional identifiable limitations. First, inclusion of infants is limited to those born after the gestational age of 28 weeks thus potentially reducing the overall incidence of NH. Severely preterm infants are at risk of severe NH and neurological impairment due to NH but survival in resource constrained settings before 28 weeks is extremely rare as ventilator assistance is unavailable. Infants who are born before 28 weeks are given supportive care and excluded from this analysis, as described and recommended by Turner et al. [21]. The estimation of gestation with ultrasound before 14 weeks of pregnancy is reliable and the quality of ultrasound service in the SMRU clinics is high [26]. If the mother attends ANC after the 24 weeks of gestational age the next best estimate will be chosen from the Dubowitz test [27] or last menstrual period if known.

A second limitation of this study is that the follow-up will last only one year and no auditory tests or magnetic resonance imaging are available. It means that the diagnosis of kernicterus will not be explored in as much detail as is possible in resource rich settings [28]. Kernicterus is most usually characterized by choreoathetoid cerebral palsy, impaired upward gaze, and sensorineural hearing loss whereas cognition is relatively spared [28]. There is also controversial discussion about the relationship between NH and developmental delay, cognitive impairment or behavioural disorders [29]. These will only become evident when the child is older than one year of age. Strict definitions of moderate and severe NH and treatment protocols will help minimize these limitations [6].

Interpretation of the data must consider the use of different types of phototherapy in the SMRU clinics (bulbs and Light-emitting diode units). Differences in irradiance will be recorded to help reduce bias in the interpretation of the results. The Bilirubin Induced Neurological Dysfunction (BIND) Score [30] has recently been validated for resource-limited settings in Africa. Unfortunately, the BIND score was not included in this study protocol. However medical staff working in the SCBU have learned to systematically describe in their ‘daily observation sheet’ the neonate’s tone, alertness, movements and sucking behaviour. This should be enough to reconstitute the BIND score, at least partially, to evaluate and categorize the neurological evolution of the neonate during their hospitalization in the SCBU.

## Conclusion

It is expected that the study will confirm that neonatal jaundice is a risk factor for neonatal mortality and long-term morbidity, and raise awareness of the problem. It will provide evidence to develop appropriate clinical and public health guidelines to address the problem, thereby improving maternal and child health care. We expect that the observations and recommendations resulting from the study can be used in other parts of South-East-Asia as well, especially in Myanmar.

## Abbreviations

ANC: Antenatal care
G6PD: Glucose-6-phosphate dehydrogenase
Hb: Haemoglobin
Hct: Haematocrit
NH: Neonatal hyperbilirubinemia
SBR: Serum bilirubin
SCBU: Special care baby unit
SMRU: Shoklo Malaria Research Unit
